# Supplementary material for: Platelet Abnormalities in Children with Laboratory-Confirmed Influenza
Source: Diagnostics (Basel). 2023 Feb 8;13(4):634. doi: 10.3390/diagnostics13040634 (PMC9954849; doi:10.3390/diagnostics13040634)
Supplement: Supplementary file 1 [file diagnostics-13-00634-s001.zip › Supplementary table S1.pdf]

Supplementary table S1. A comparison of platelet parameters based upon gender for the whole study group and within the age groups; the p value for statistically significant differences is marked in red.

whole group	MALE			FEMALE			
	median	LQ	UQ	median	LQ	UQ	p
Platelet count [$10^3/\mu L$]	238.000	182.000	306.000	250.500	191.500	329.500	0.075146
MPV [fL]	9.800	9.300	10.300	9.600	9.100	10.200	0.042047
MPV/PLT	0.041	0.031	0.054	0.040	0.028	0.051	0.066924
PLT/LYM	104.217	65.054	181.333	94.208	62.552	146.380	0.229414
<1yo							
Platelet count [$10^3/\mu L$]	302.500	247.000	407.500	356.000	246.000	451.000	0.169036
MPV [fL]	9.700	9.300	10.200	9.900	9.200	10.400	0.358308
MPV/PLT	0.032	0.024	0.040	0.026	0.020	0.042	0.175121
PLT/LYM	93.930	59.787	138.935	72.881	47.303	122.848	0.066581
1-2yo							
Platelet count [$10^3/\mu L$]	256.000	205.000	336.000	291.500	207.000	335.000	0.480631
MPV [fL]	10.000	9.100	10.300	9.400	8.900	9.900	0.045900
MPV/PLT	0.039	0.028	0.047	0.033	0.026	0.047	0.407714
PLT/LYM	80.308	45.567	179.487	78.829	58.367	104.762	0.801471
2-5yo							
Platelet count [$10^3/\mu L$]	234.000	174.000	292.000	223.000	178.000	287.000	0.833156
MPV [fL]	9.700	9.100	10.300	9.500	9.100	10.000	0.419820
MPV/PLT	0.041	0.032	0.059	0.044	0.034	0.054	0.906376
PLT/LYM	97.030	57.178	186.885	93.706	67.982	138.129	0.956603
>5yo							
Platelet count [$10^3/\mu L$]	192.500	161.000	236.000	218.000	181.000	264.000	0.026372
MPV [fL]	10.000	9.500	10.500	9.800	9.300	10.500	0.088770
MPV/PLT	0.053	0.041	0.065	0.045	0.038	0.057	0.048902
PLT/LYM	136.323	90.593	205.956	134.568	101.208	222.115	0.559909
