# Supplementary material for: Platelet Abnormalities in Children with Laboratory-Confirmed Influenza
Source: Diagnostics (Basel). 2023 Feb 8;13(4):634. doi: 10.3390/diagnostics13040634 (PMC9954849; doi:10.3390/diagnostics13040634)
Supplement: Supplementary file 1 [file diagnostics-13-00634-s001.zip › Supplementary table S2.pdf]

Supplementary table S2. A correlation between platelet parameters (platelet count- PLT, mean platelet volume- MPV, mean platelet volume/platelet count ratio- MPV/PLT, platelet to lymphocyte ratio- PLT/LYM) and hydration status parameters (pH, HCO_3, and base excess) for the whole study group and age subgroups; the results from Spearmann's rank correlation test. The results are showed as correlation coefficients, statistically significant results are marked in red. Abbreviations: yo- years old, BE- base excess.

.

WHOLE GROUP	pH	HCO_3	BE
Platelet count $[10^3/\mu L]$	-0.06	-0.15	-0.17
MPV [fL]	0.08	0.09	0.12
MPV/PLT	0.08	0.14	0.17
PLT/LYM	-0.08	0.05	0.02

UNDER 1YO	pH	HCO_3	BE
Platelet count $[10^3/\mu L]$	-0.16	0.09	0.03
MPV [fL]	0.14	0.14	0.24
MPV/PLT	0.18	-0.08	0.00
PLT/LYM	-0.01	0.16	0.17

1-2YO	pH	HCO_3	BE
Platelet count $[10^3/\mu L]$	-0.18	0.06	-0.07
MPV [fL]	0.07	-0.08	0.01
MPV/PLT	0.17	-0.08	0.06
PLT/LYM	0.18	-0.01	0.09

2-5YO	pH	HCO_3	BE
Platelet count $[10^3/\mu L]$	-0.12	-0.27	-0.27
MPV [fL]	0.03	0.04	0.03
MPV/PLT	0.12	0.25	0.25
PLT/LYM	-0.19	-0.18	-0.23

>5YO	pH	HCO_3	BE
Platelet count $[10^3/\mu L]$	-0.07	-0.11	-0.12
MPV [fL]	0.08	0.06	0.09
MPV/PLT	0.08	0.05	0.06
PLT/LYM	-0.15	-0.12	-0.16
