# Supplementary material for: Platelet Abnormalities in Children with Laboratory-Confirmed Influenza
Source: Diagnostics (Basel). 2023 Feb 8;13(4):634. doi: 10.3390/diagnostics13040634 (PMC9954849; doi:10.3390/diagnostics13040634)
Supplement: Supplementary file 1 [file diagnostics-13-00634-s001.zip › Supplementary table S3.pdf]

Supplementary table S3. A comparison of hydration status parameters (pH, HCO_3, and base excess) between the groups with presence (vs absence) of platelet count abnormalities, thrombocytosis, thrombocytopaenia. The comparisons for the whole study group and age subgroups are presented; statistically significant results are marked in red. Abbreviations: yo- years old, BE- base excess.

	WHOLE GROUP<br>ABNORMAL PLATELET COUNT<br>YES			WHOLE GROUP<br>ABNORMAL PLATELET COUNT<br>NO			
	median	LQ	UQ	median	LQ	UQ	p
pH	7.43	7.40	7.47	7.43	7.41	7.46	0.5747
HCO_3	22.50	21.20	24.20	21.70	19.70	23.30	0.0020
BE	-1.10	-2.20	0.10	-2.00	-3.50	-0.40	0.0042

	UNDER 1YO<br>ABNORMAL PLATELET COUNT<br>YES			UNDER 1YO<br>ABNORMAL PLATELET COUNT<br>NO			
	median	LQ	UQ				p
pH	7.42	7.39	7.46	7.44	7.42	7.47	0.0600
HCO_3	22.20	20.70	24.00	21.30	19.10	23.00	0.1531
BE	-1.40	-3.10	-0.50	-2.20	-3.70	-0.30	0.4065

	1-2YO<br>ABNORMAL PLATELET COUNT<br>YES			1-2YO<br>ABNORMAL PLATELET COUNT<br>NO			
	median	LQ	UQ	median	LQ	UQ	p
pH	7.42	7.39	7.51	7.44	7.41	7.46	0.7607
HCO_3	21.20	20.45	22.80	19.65	18.60	21.85	0.0789
BE	-1.95	-3.45	-0.35	-3.40	-4.75	-1.40	0.1671

	WHOLE GROUP<br>THROMBOCYTOSIS<br>YES			THROMBOCYTOSIS<br>NO			
pH	7.42	7.39	7.47	7.43	7.41	7.46	0.1836
HCO_3	21.60	20.70	24.10	21.80	19.90	23.50	0.3911
BE	-1.60	-3.10	-0.20	-1.80	-3.30	-0.30	0.7477

	UNDER 1YO<br>THROMBOCYTOSIS<br>YES			THROMBOCYTOSIS<br>NO			
pH	7.42	7.39	7.45	7.44	7.42	7.47	0.0182
HCO_3	21.80	20.40	24.05	21.40	19.10	23.00	0.2521
BE	-1.60	-3.10	-0.60	-2.10	-3.65	-0.35	0.7043

	1-2YO<br>THROMBOCYTOSIS<br>YES			THROMBOCYTOSIS<br>NO			
pH	7.42	7.39	7.47	7.44	7.40	7.46	0.5778
HCO_3	21.20	20.60	23.60	19.85	18.60	21.90	0.1243
BE	-2.40	-3.50	-0.20	-3.15	-4.70	-1.30	0.4040

	WHOLE GROUP<br>THROMBOCYTOPAENIA<br>YES			THROMBOCYTOPAENIA<br>NO			
pH	7.43	7.41	7.47	7.43	7.41	7.46	0.5581
HCO_3	22.80	22.00	24.20	21.65	19.80	23.40	0.0009
BE	-0.65	-1.60	0.20	-1.95	-3.50	-0.40	0.0004

	UNDER 1YO<br>THROMBOCYTOPAENIA<br>YES			THROMBOCYTOPAENIA<br>NO			
pH	7.46	7.43	7.49	7.43	7.41	7.47	0.3128
HCO_3	22.60	22.20	23.00	21.40	19.30	23.30	0.3870
BE	-0.80	-0.90	-0.40	-2.10	-3.50	-0.40	0.2203

	1-2YO<br>THROMBOCYTOPAENIA<br>YES			THROMBOCYTOPAENIA<br>NO			
pH	7.47	7.39	7.55	7.44	7.40	7.46	0.7404
HCO_3	21.15	20.30	22.00	20.00	18.60	21.90	0.4661
BE	-1.50	-2.50	-0.50	-3.30	-4.70	-1.30	0.2332

2-5YO

ABNORMAL PLATELET COUNT

	YES			NO			
	median	LQ	UQ	median	LQ	UQ	p
pH	7.44	7.42	7.47	7.43	7.40	7.45	0.3387
HCO_3	22.95	21.65	24.70	21.50	19.90	22.80	0.0063
BE	-0.40	-1.90	0.35	-2.10	-3.50	-0.90	0.0033

THROMBOCYTOSIS

	YES			NO			
pH	7.46	7.42	7.47	7.43	7.40	7.45	0.4673
HCO_3	21.70	21.20	25.70	21.70	19.90	22.90	0.3043
BE	-1.00	-2.00	-0.10	-2.00	-3.40	-0.60	0.1528

THROMBOCYTOPAENIA

	YES			NO			
pH	7.43	7.42	7.47	7.43	7.40	7.45	0.5223
HCO_3	23.30	22.40	24.60	21.50	19.90	22.80	0.0137
BE	-0.20	-1.80	0.50	-2.10	-3.40	-0.90	0.0140

>5YO

ABNORMAL PLATELET COUNT

	YES			NO			
	median	LQ	UQ	median	LQ	UQ	p
pH	7.43	7.41	7.45	7.42	7.41	7.45	0.9344
HCO_3	23.85	21.70	25.00	22.70	21.20	24.40	0.2202
BE	-0.90	-1.60	0.60	-1.00	-2.40	0.40	0.2604

THROMBOCYTOSIS

	YES			NO			
pH				7.43	7.41	7.45	1.0000
HCO_3				22.80	21.45	24.40	1.0000
BE				-1.00	-2.20	0.40	1.0000

THROMBOCYTOPAENIA

	YES			NO			
pH	7.43	7.41	7.45	7.42	7.41	7.45	0.9344
HCO_3	23.85	21.70	25.00	22.70	21.20	24.40	0.2202
BE	-0.90	-1.60	0.60	-1.00	-2.40	0.40	0.2604
